# SARS-CoV-2 and the nervous system: current perspectives

**DOI:** 10.1007/s00705-023-05801-x

**Published:** 2023-06-01

**Authors:** Amrita Pattanaik, Sushma Bhandarkar B, Lonika Lodha, Srilatha Marate

**Affiliations:** 1 Manipal Institute of Virology, Manipal Academy of Higher Education (MAHE), PIN-576104 Manipal, Karnataka India; 2 Department of Neurovirology, National Institute of Mental Health and Neurosciences (NIMHANS), PIN-560029 Bengaluru, Karnataka India

**Keywords:** SARS-CoV-2, COVID-19, Neurological impairment, Neuro COVID, Post-COVID-19 sequelae

## Abstract

SARS-CoV-2 infection frequently causes neurological impairment in both adults and children. Recent publications have described significant aspects of the viral pathophysiology associated with neurological dysfunction. In theory, neurological manifestations following SARS-CoV-2 infection may be caused directly by the effects of the virus infecting the brain or indirectly by the local and systemic immune responses against the virus. Neurological manifestations can occur during the acute phase as well as in the post-acute phase of the infection. In this review, we discuss recent literature describing the association of nervous system disorders with COVID-19.

**Supplementary Information:**

The online version contains supplementary material available at 10.1007/s00705-023-05801-x.

## Introduction

From the time COVID-19 was declared a pandemic by the World Health Organization (WHO), clinicians began observing neurological manifestations of both mild and severe intensity in acutely ill patients with confirmed infection [1–3]. Early retrospective studies from China and France revealed that a very large number of COVID-19 patients had experienced neurological complications during the period of their hospitalization [4, 5]. However, since these reports were limited to hospitalized patients, they are not reflective of the true community-wide burden of neurological manifestations following SARS-CoV-2 infection. Furthermore, since the data used in many of these studies were extracted from the hospitals’ electronic records, there is a strong possibility that some nonspecific neurological symptoms were overlooked [6]. Considering the magnitude of the pandemic, there is a strong likelihood of neurological manifestations being experienced by a much larger population of COVID-19 patients during the course of their illness than what has been reported [7]. According to the published literature, neurological complications, such as cognitive dysfunction and encephalopathy, appear to be more debilitating than complications reported in other organ systems in COVID-19 [8]. The spectrum of neurological manifestations in SARS-CoV-2 infection likely represents multiple pathogenic pathways. Various mechanisms leading to the development of the neurological manifestations following neurotropic invasion have been proposed, including endothelial dysfunction, hyperinflammation, hypercoagulability, hypoxia, and general critical illness. There is still much to be explored in order to fully comprehend the pathogenicity of SARS-CoV-2 and its deleterious effects on the nervous system [7, 9].

Neurological involvement has been found at different stages of SARS-CoV-2 infection – during acute infection and as post-acute sequelae manifesting in a chronic course of infection [9]. Among the many reported, the most common neurological symptoms associated with COVID-19 have been anosmia, encephalopathy, and stroke [10]. In the acute phase, infected patients frequently show nonspecific symptoms such as generalized weakness, dizziness, headache, nausea, anosmia, and dysgeusia [11]. Neurological manifestations are also frequently seen in ‘long COVID’ syndrome. Anosmia and dysgeusia, as well as neuropsychiatric symptoms, have been reported to persist for months following infection [12].

In this review, we highlight the literature focusing on clinical observations that suggest associations between SARS-CoV-2 infection and the nervous system. We also discuss the different mechanisms of neural injury that lead to various complications. Knowledge about the possible neurological manifestations of COVID-19 is vital for physicians to recognize, treat, and manage complications of the nervous system.

## Methods

We searched the PubMed database for literature published between December 1, 2019, and April 1, 2023. The following search terms were included: “COVID-19”, “SARS-CoV-2”, “neuroCOVID”, “encephalopathy”, “neurological impairment”, “neurological deterioration”, “encephalitis”, “neurological manifestations”, “post-COVID symptoms”, “long COVID”, and “neurological symptoms”. Observational and interventional studies involving adult subjects were included. Commercial reports and government publications and reports were not included. Information about disease pathophysiology and clinical manifestations was extracted from the included studies.

The results are reported in a narrative form under the headings Pathogenesis: infection and neuroinflammation and Neurological manifestations. Patient-related clinical outcomes, wherever available, were also included in this summary.

## Pathogenesis: infection and neuroinflammation

SARS-CoV-2, the seventh known human coronavirus, is a single-stranded enveloped RNA virus. It shares 79.5% genome sequence identity with SARS-CoV. It also shares 89–96% nucleotide sequence identity with bat coronaviruses [6]. SARS-CoV-2 binds to its receptor, angiotensin converting enzyme 2 (ACE-2), an important component of the renin-angiotensin system, to initiate replication in its host cells. The formation of the SARS-CoV-2/ACE-2 complex leads to the activation of transmembrane protease, serine 2 (TMPRSS2), which then cleaves the spike protein, allowing the SARS-CoV-2/ACE-2 complex to be internalized into the cell by endocytosis [13]. An alternate co-receptor for the virus is the membrane protein neuropilin 1 (NRP 1) [14]. After uncoating within the cell, the viral genome is used as an mRNA for translation of the viral non-structural proteins, forming a replicase-transcriptase complex (RTC) that produces subgenomic RNA for translation of the viral structural proteins. After assembly, new virions are released by exocytosis [15].

### Direct invasion of the nervous system

#### Via olfactory nerves

**Fig. 1:**
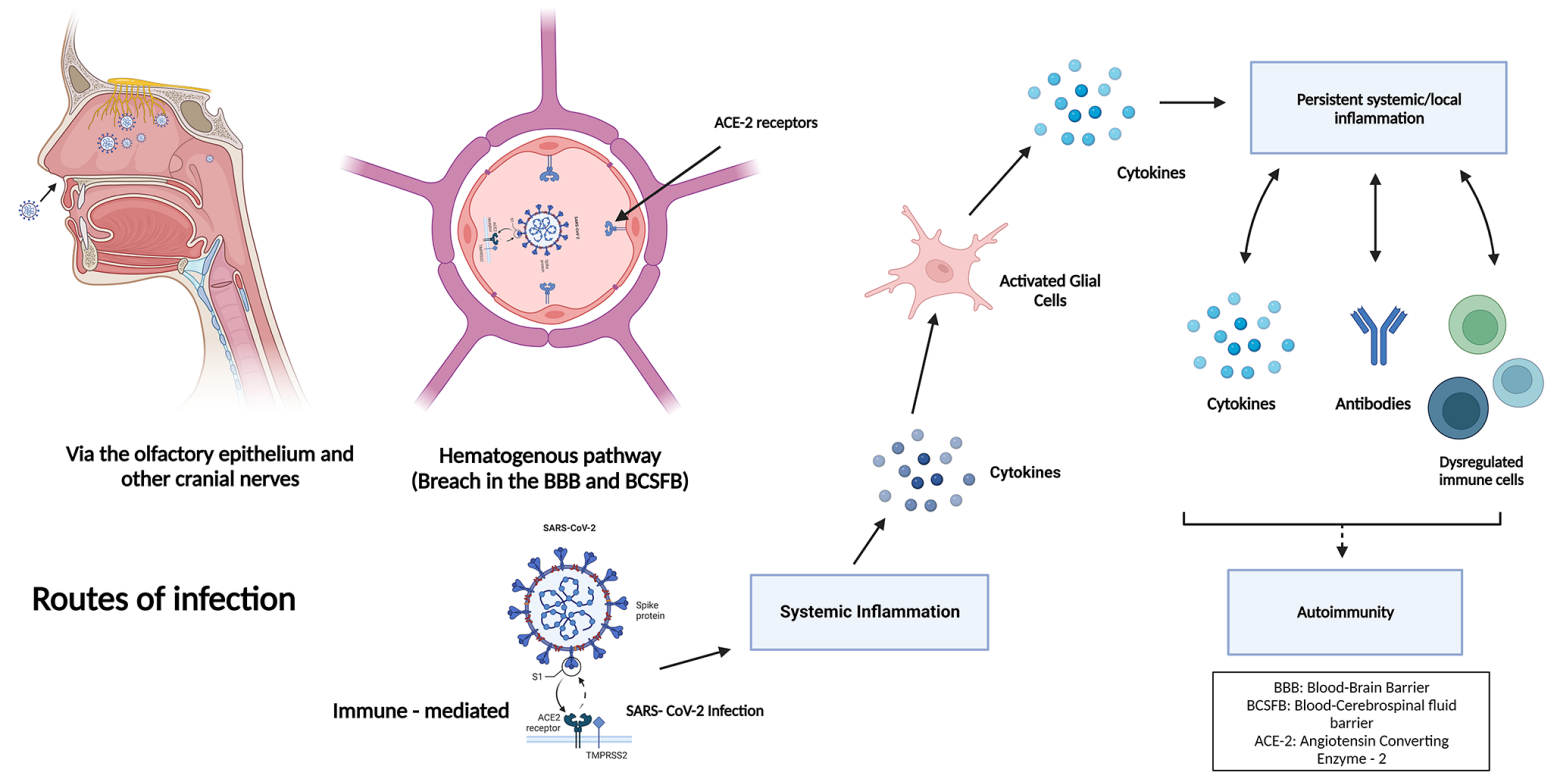
Potential routes of SARS-CoV-2 entry into the CNS (created using BioRender). A potential entry route of SARS-CoV-2 into the CNS could be via the olfactory epithelium. Another pathway of entry could be by infection of the brain capillary endothelium (hematogenous pathway). Immune responses to viral infection may result in disruption of the blood brain barrier, resulting in the creation and maintenance of an inflammatory environment in the CNS.

The chemosensory loss seen in COVID-19 patients in the form of anosmia, ageusia, or dysgeusia can be attributed to dysfunctional or damaged olfactory and gustatory receptors and their supporting cells or disruption of interactions with semaphorins (key molecules in olfactory and gustatory signalling pathways) [16]. There is no evidence of the expression of ACE-2 in the olfactory nerve, which seems to rule out direct neuronal damage by the virus as the cause of anosmia. However, ACE-2 receptors have been demonstrated in the olfactory mucosa by immunostaining. Olfactory epithelial sustentacular cells have been shown to express ACE-2. This has been demonstrated by single-cell sequencing and confirmed by immunostaining [17–19]. The spike protein (detected by immunohistochemistry) and RNA (detected by real-time PCR) of SARS-CoV-2 virus have been demonstrated in olfactory mucosa of post-mortem samples from SARS-CoV-2 infected individuals [20–22]. Infection of the olfactory epithelium (Fig. 1) can therefore account for the anosmia seen in the disease and potentially serve as a pathway of entry of the virus into the central nervous system (CNS) [18, 19].

#### Via other cranial nerves

Other cranial nerves, such as the vagus, glossopharyngeal, and trigeminal nerves, may also be potential routes for the virus to enter the brain via retrograde axonal transport. These nerves get exposed to the virus during the course of infection. The vagus nerve, which is connected to the gastrointestinal tract as a part of the enteric nervous system, has an abundance of ACE-2 and NRP 1 receptors [23]. Some studies have suggested that the virus can access the CNS through peripheral fibers of the vagus nerve in the lung, similar to influenza virus [24, 25]. A study examining brainstem neuropathology demonstrated the presence of SARS-CoV-2 in vagus nerve fibers by the use of immunohistochemistry (IHC) [26]. Another study examining the vagal and human glossopharyngeal nerves at the level of the medulla oblongata showed that ACE-2 receptors and neuropilin 1 (NRP1) co-receptors are widely expressed in axons, myelin sheaths, and nerve bundles. Together with ACE-2 and NRP1, the presence of TMPRSS2 in the supportive cells of the vagal and glossopharyngeal nerves has also been demonstrated [14]. SARS-CoV-2 dissemination has been seen in the trigeminal nerve as well, which has been implicated in anosmia and headache in patients. One hypothesis states that the virus can enter the CNS by invading the sensory axon of the trigeminal nerve in the nasal cavity. One study showed a high level of SARS-CoV-2 RNA in the trigeminal ganglion in deceased COVID-19 patients, and another post-mortem study showed axonal degeneration and cell loss in the trigeminal nerve [17, 27]. Despite this evidence of the direct involvement of nerves in the disease process, more studies on the pathophysiology of COVID-19 need to be undertaken.

### Indirect invasion of the nervous system

#### Hematogenous route

A pathway that is potentially important for invasion of the CNS is infection of the brain capillary endothelium, which forms the neurovascular unit of the blood brain barrier (BBB). Studies examining post-mortem brain samples have shown the presence of virus-like particles in the capillary endothelium of the brain. The presence of SARS-CoV-2 nucleic acid in the brain has been demonstrated by polymerase chain reaction (PCR) targeting different regions of the viral genome [28]. The choroid plexus epithelial cells that form a part of the blood-cerebrospinal fluid (BCSF) barrier might also be an entry point, as evidenced in human brain organoids [29]. Evidence for the presence of the spike protein has also been found in the choroid plexus vasculature by immunostaining with anti-spike protein antibody, and by PCR. It has been seen that the infection is restricted to the lumina of the choroid plexus capillaries and medium-sized blood vessels [30].

#### Immune-mediated mechanisms

Immune-mediated mechanisms have been shown to play a significant role in neuroinvasion by SARS-CoV-2. Systemic inflammatory responses to infection are responsible for triggering the activation of microglial cells by excessive production of proinflammatory cytokines, including interleukins (IL-6, IL-2, IL-12, and IL-15) and tumour necrosis factor alpha (TNF-α) [9]. Some proinflammatory cytokines have been shown to have saturable mechanisms of transport from the blood to the CNS. It has been demonstrated that blood-borne proinflammatory cytokines can disrupt and traverse the blood brain barrier (BBB) to reach the cerebrospinal fluid and interstitial fluid spaces of the brain and spinal cord [31]. They, thus, play an important role in the development of neurological symptoms in patients. An increase in the levels of intrathecal interleukins (IL- 6, IL-18, IL-15) and macrophage inflammatory protein 1β (MIP-1β) has been seen in a subset of immunocompetent COVID-19 patients displaying neurological manifestations when compared to a control group of immunosuppressed SARS-CoV-2-infected patients who did not have any neurological symptoms [32]. Analysis of the CSF of a SARS-CoV-2-infected individual diagnosed with acute encephalopathy showed increased levels of pro-inflammatory cytokines, including monocyte chemoattractant protein 1 (MCP-1) [33]. Elevated levels of MCP-1 have been seen in other neuroinfectious or neuroinflammatory disorders such as neuroAIDS, bacterial meningitis, and multiple sclerosis [34–36].

### Neuroinflammation

Neuroinflammation involves different inflammatory responses elicited against particular stimuli in the CNS. This is a result of a multitude of reactive components of the neurovascular unit (NVU) and their responses. These components include neurons, microglia, astrocytes, oligodendrocytes, and endothelial cells [37].

**Fig. 2:**
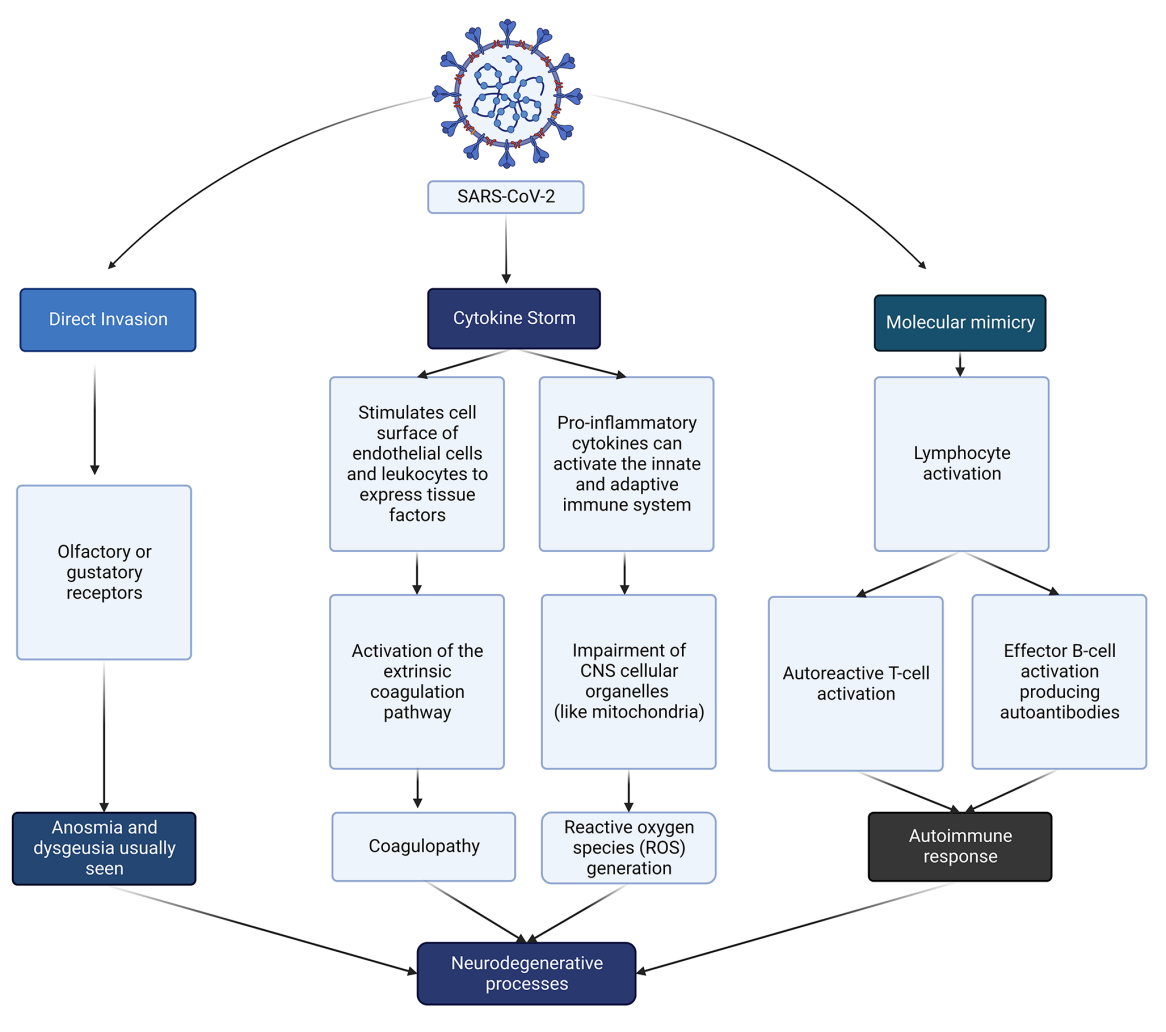
Pathogenesis of neurodegenerative processes in COVID-19 (created using BioRender). Neurodegenerative processes can be observed as a result of direct invasion via the olfactory or gustatory receptors, coagulopathy, generation of reactive oxygen species (ROS), or induction of autoimmunity.

The various inflammatory mediators that are produced by these activated components include cytokines, chemokines, and free radicals (Fig. 2). These mediators contribute to the increased permeability and infiltration of immune cells across the BBB, thereby promoting neuroinflammation [38–40].

It has been found that SARS-CoV-2-infected patients with acute neurological complications have elevated levels of proinflammatory cytokines such as IL-6, IL-18, and IL-8 when compared to healthy controls [41]. Similarly, another study reported increased levels of proinflammatory cytokines (IL-6, IL-8, and TNF-α) in CSF from a SARS-CoV-2-infected individual with akinetic mutism. It was also shown that cytokine levels decreased with the recovery of the individual [42]. An *in vitro* study in which human brain microvascular endothelial cells (BMVECs) were exposed to SARS-CoV-2 spike protein showed that there was an association between a reduction in the expression of tight-junction and an increase in the levels of the cytokines IL-6, IL-10, and TNF-α [43].

Other studies have shown that, in anosmia, the loss of olfactory function could be correlated with infection of the olfactory epithelium and increased expression of cytokines such as IL-6 [10, 44]. The regeneration of nasal epithelial cells may be compromised, as the olfactory mucosa is sensitive to cytokines [45]. Proinflammatory cytokines such as IL-6 can potentially activate Toll-like receptors, which may lead to inflammation of taste buds and, thereby, a loss of taste [46, 47].

Oxidative stress, a significant inflammatory response that has been implicated in neuroAIDS in human immunodeficiency virus (HIV) infection, is suggested to play a role in the pathogenesis of neurological manifestations of COVID-19. An *in vitro* study performed on human microglia treated with the SARS-CoV-2 spike protein showed that there was an increase in mitochondrial respiration, leading to the production of ROS, and increased oxidative stress arising as a result of an imbalance between ROS production and the body’s ability to detoxify the reactive intermediates. This might contribute to the neurodegenerative process [48–50]. Neurodegenerative disease may also occur due to the activation of autoimmune responses by molecular mimicry of self-antigens by viral antigens. An example of this is Guillain-Barre Syndrome (GBS), an autoimmune disease that has been seen in some COVID-19 patients [51, 52].

Coagulopathy has been observed in patients with severe COVID-19. The onset of stroke in COVID-19 could be due to hypercoagulability and vasculitis. In acute infections, mediators of inflammation such as tissue factors may induce hypercoagulation [46]. Tissue factors can act as receptors for factor VII. The expression of tissue factor on the cell surface of endothelium and leukocytes can be stimulated by proinflammatory cytokines such as TNF-α by the extrinsic coagulation pathway. A prothrombotic state and vasculitis could be attributed to elevation of levels of adhesion molecules, cytokines, angiotensin II, and D-dimer and a decrease in fibrinolysis, which has been associated with the disseminated intravascular coagulation (DIC) seen in severe COVID-19 [47, 53]. Additionally, the proinflammatory cytokine IL-6 has been shown to be responsible for stimulation of platelet production, tissue factor gene expression, and fibrinogen production by endothelial and monocyte cell types [54]. Furthermore, damage to endothelial cells by cytokine storm can lead to the production of phosphatidylserine (PS), which can promote thrombin production. Release of plasminogen activator inhibitor type 1 (PAI-1) by damaged endothelial cells can inhibit the fibrinolytic system, leading to thrombosis [55]. Systemic coagulopathy and vasculopathy result in neurological manifestations such as encephalopathy and delirium seen in a SARS-CoV-2 infection [10].

The activation of glial cells (astrocytes, oligodendrocytes, and microglial cells) due to systemic infection modulates neuroinflammatory responses [37]. Microglial cells, when activated, polarise to the M1 phenotype. This phenotype mediates a proinflammatory response which involves an increase in proinflammatory mediators such as TNF-α, IL-β, IL-6, and reactive oxygen species (ROS) [37, 56]. This proinflammatory state can lead to the activation of astrocytes [37] or lead to the destruction of microglia [56]. A post-mortem study done on the brainstems and olfactory bulbs of individuals who had succumbed to SARS-CoV-2 infection revealed high microglial immune activation with microglial nodules and immune cell clusters (such as CD8^+^ T cells) associated with axonal damage [57]. Activated microglia can produce IL-1 and TNF-α, which can activate astrocytes, which then produce inflammatory factors such as TNF-α, nitric oxide (NO), and ROS. This mutual interaction between microglia and astrocytes amplifies neuroinflammation [37, 58]. Another stress factor that can contribute to disruption of the integrity of the BBB is hypoxia, which can lead to the infiltration of immune cells and proinflammatory cytokines into the brain [51]. Neurological manifestations such as stroke and meningoencephalitis may be due the cytokine storm (Fig. 2) induced by SARS-CoV-2 infection [46].

## Neurological manifestations of SARS-CoV-2

### Parainfectious/acute neurological manifestations

Various acute neurological manifestations have been associated with COVID-19 (Table 1). The most common nonspecific symptoms of the nervous system reported in COVID-19 patients include olfactory and gustatory dysfunction presenting as anosmia, dysgeusia, headache, and fatigue. A meta-analysis of 350 studies with 145,721 subjects found that the pooled prevalence of taste and smell dysfunction were 21% and 19%, respectively [59]. In most cases, these symptoms were the initial manifestations of the illness and were not associated with nasal discharge or congestion. However, taste and smell disturbances were rarely the only COVID-19 symptoms and were accompanied by other manifestations. Anosmia has been reported more commonly in younger individuals than in older ones. Also, it has been seen more frequently in women than in men [60]. Smell and taste disorders resolve spontaneously without the requirement for any specific management in most patients with COVID-19. However, 10–20% of these patients have serious or long-term deficits [59, 61].

**Table 1:** Prevalence of various acute neurological manifestations included in meta-analyses

Year of publication	Number of studies analysed	Anosmia	Dysgeusia	Headache	Other cranial nerve dysfunction	Fatigue	Encephalopathy	Myalgia	Encephalitis and meningitis	Cerebrovascular disease	Seizures	Psychiatric disorders	Reference
2022	20	20.8	23.9	24.2	8.6* 5.6**	26.3	33.7	26.7	4.1	29.6	7.7	41.7 (Delirium)	[62]
2021	350	19	21	13	-	32	-	20	-	2	-	24	[59]
2021	240	26.4	27.2	14.6	-	33.6	23.5	21.4	0.6	9.9	4	14.2	[63]
2021	147	43.1	37.2	20.7	3* 3**	37.8	-	25.1	-	1.6	0.06	23 (Depression) 15.9 (Anxiety)	[64]
2021	168	33	33	29	6^*^	-	26	33	2	12	4	-	[65]
2021	44	18.3	19.6	12.1	-		9.4	22.2	-	2.5	-	-	[66]
2021	58	25.3	35.4	10.1	-	42.9	-	-	-	4.3	-	-	[67]
2020	41	35.7	33.3	-	-	33.2	-	-	-	-	-	-	[68]
2020	74	35.8	38.5	14.7	-	-	-	19.3	-	2.3	-	-	[69]
2020	51	35	33	12	-	-	-	19	-	3	-	-	[70]

The frequencies of acute neurological manifestations are presented as percentages

*, Vision impairment; **, hearing impairment

Headache, with an incidence of 25–47%, has been reported as an acute-phase manifestation of COVID-19. Fatigue was another common, debilitating symptom during acute illness. The reported prevalence was around 27–32%. However, it is likely to have been underdiagnosed due to the subjectivity of reporting [71–73].

Other reported cranial nerve dysfunctions in COVID-19 patients include oculomotor dysfunction, hearing loss, facial palsy, ocular neuropathies, and lower cranial-nerve abnormalities. During or after infection, sudden sensorineural hearing loss (unilateral or bilateral) with an incidence of 13% and persistent tinnitus with an incidence of 15% have been reported relatively frequently [71, 74].

Another nonspecific neurological manifestation commonly seen in COVID-19 patients is myalgia, which has been documented and reported in 22–63% of patients. It has been reported in mild as well as severe COVID-19 [61]. Increased levels of creatinine kinase (CK) have been seen in more-severe cases. Myopathy has also been reported in some patients during the acute phase of infection [60]. It is not clear if this is due to the direct effect of the virus on myocytes or due to the local and/or systemic immune response against the invading virus [75].

Among the more-specific neurological symptoms, encephalopathy is commonly diagnosed in patients with COVID-19. Different studies have shown its prevalence to be 8%-12% [62, 76]. The term “encephalopathy” includes altered consciousness, delirium, agitation, confusion, or coma. Signs of encephalopathy are seen in most critically ill COVID-19 patients. Some of the risk factors associated with encephalopathy are older age, smoking, prior history of neurological derangement, diabetes, chronic kidney disease, cerebral vasculitis, dyslipidaemia, cardiac failure, and hypertension. Of the patients admitted to an intensive care unit (ICU), 60% present with agitation and delirium. In patients older than 60 years, acute confusion or delirium was seen with a pooled prevalence of 34% and was associated with higher mortality [9, 59]. Occasional cases of encephalitis and meningitis have also been reported in COVID-19 patients. The incidence of encephalitis in COVID-19 is less than 1% but can be as high as 6–7% in severe disease [77]. Patients with encephalitis or meningitis due to suspected SARS-CoV-2 infection have presented with a wide range of typical presentations (signs of meningeal irritation, altered sensorium) and atypical presentations (like seizure, akinetic mutism, psychosis, oculocephalic reflex, catatonia, coma) [42, 78–82].

New-onset seizures are one of the important acute-phase manifestations of neurological dysfunction reported during SARS-CoV-2 infection. A recent study concluded that acute seizures occurred in less than 5% of the hospitalized COVID-19 patients [72]. Acute stroke or encephalitis are frequently associated with seizures. Various studies have concluded that most of these seizures developed in the absence of a prior diagnosis of epilepsy [83].

An association between COVID-19 and cerebrovascular disease has been convincingly demonstrated, with manifestations including ischemic stroke, intracerebral thrombosis, and intracerebral haemorrhage. These manifestations have been observed not only in older patients with multiple significant cerebrovascular risk factors but also in young patients without any comorbidities. A meta-analysis involving 18,258 COVID-19 patients showed that the pooled prevalence of cerebral ischemia was 2.9% and that of cerebral thrombosis was 2.2% [62]. Morassi et al., in a case series, reported biochemical evidence of coagulopathy in more than 65% of patients with COVID-19 [84]. Stroke usually developed within a month of onset of the symptoms of COVID-19. In different studies, it was seen that SARS-CoV-2 infection was an independent risk factor for stroke in hospitalized patients [85–88].

Psychiatric disorders have been one of the significant CNS disturbances described during the pandemic. A study from the United States retrospectively reported psychiatric manifestations in COVID-19 patients within the first 3 months of infection. These patients did not have any previous history of psychiatric disorders. The most frequent disorders reported in that study were insomnia, anxiety, and dementia. Ten to 38% of cases of depression and/or anxiety associated with SARS-CoV-2 infection occurred during the acute phase of illness. In 5–13% of the cases, symptoms persisted even after the resolution of the infection [71–73, 89].

### Post-acute neurological manifestations

A few months into the SARS-CoV-2 pandemic, anecdotal reports from survivors of the illness started to emerge from social media and patient support groups, complaining of non-resolution of symptoms or protracted course of illness for weeks to months [90]. In the initial reports, the constellation of persistent symptoms in COVID-19 survivors was labelled as “long-haul COVID” or “long-tail COVID” by the mainstream media. It was reported that after the resolution of acute respiratory and febrile illness, patients suffered from a wide spectrum of systemic and organ-system-specific symptoms that persisted long after microbiological recovery (i.e., a negative PCR test) [91].

However, research data were scarce at that time, and there was no concrete definition or diagnostic criteria for this syndrome. Subsequently, in October 2021, the WHO formally recognised this post-COVID-19 condition by formulating a clinical case definition. The definition states that

*Post COVID-19 condition occurs in individuals with a history of probable or confirmed SARS-CoV-2 infection, usually 3 months from the onset of COVID-19 with symptoms that last for at least 2 months and cannot be explained by an alternative diagnosis. Common symptoms include fatigue, shortness of breath, cognitive dysfunction but also others and generally have an impact on everyday functioning. Symptoms may be new onset following initial recovery from an acute COVID-19 episode or persist from the initial illness. Symptoms may also fluctuate or relapse over time*.

Additionally, a code was assigned in the tenth revision of the International Classification of Diseases (ICD-10) for this condition [92].

Currently, several different terms are being used for post-COVID-19 conditions, such as post-acute sequelae of SARS-CoV-2 infection (PASC), post-acute COVID-19 syndrome (PACS), long COVID, persisting COVID, and post-COVID syndrome, among others [12]. The post-acute manifestations of COVID-19 are quite diverse, including, but not limited to, systemic, neurological, cardiovascular, respiratory, gastrointestinal, renal, immunological, and musculoskeletal dysfunctions [93].

#### Prevalence and duration of long COVID

The overall global prevalence of PACS has been reported to be 0.37 at 30 days, 0.25 at 60 days, 0.32 at 90 days, and 0.49 at 120 days postinfection, according to a recent meta-analysis [94]. Higher prevalence rates of 63.2% at 30 days, 71.9% at 60 days, and 45.9% at ≥ 90 days after onset of illness have also been reported [95].

In a large electronic health record review of more than 200,000 patients diagnosed with COVID-19, it was found that 33.62% of these patients presented with neurological or psychiatric sequelae within 6 months of acute infection [96]. It is important to note that there may be substantial overlap of acute and post-acute neurological manifestations of COVID-19. Therefore it is important to exclude acute infection in order to identify post-infectious sequelae [12].

The average duration from acute COVID-19 infection to post-infectious neurological sequelae has been found in a meta-analysis of 55 such cases to be 33.2 days. The conditions reported to occur, in descending order of frequency, were Guillain-Barre Syndrome (GBS), stroke, optic neuritis, and encephalitis. Less frequently, transverse myelitis, neuromyopathy/neuropathy, encephalopathy, Parkinsonism, status epilepticus, Bell’s palsy, vestibulocochlear neuritis, opsoclonus myoclonus syndrome, and myopathy were also reported to occur [97].

Studies have been carried out to evaluate the duration of persistence of long COVID symptoms, and it has been demonstrated that 37.8% of the patients studied experienced symptoms until the end of one year following acute infection [98]. However, the proportion of survivors with sequelae has been shown to decrease over time, from 68% at 6 months postinfection to 55% at two years postinfection. At the end of two years, the health status of these patients was seen to deteriorate considerably compared to the general population [99]. This long-persisting illness, i.e., at 12–18 months postinfection, has been dubbed “very long COVID” in a study with a reported prevalence of 61% in the 121 patients studied [100].

Several investigators have compared the incidence of persistent manifestations in COVID-19 survivors with other control groups, leading to disparate conclusions. A significantly higher prevalence of persistent symptoms or worse health outcomes has been demonstrated in COVID-19 patients when compared to uninfected controls, influenza-virus-infected controls, and controls with other respiratory tract infections [96, 101, 102]. In contrast, a few investigators have found no evidence of varying recovery rates in olfactory dysfunction in COVID-19 PCR-positive and PCR-negative patients [103]. Also, no significant difference in the prevalence of neurological and cognitive deficits has been seen in COVID-19 cases and uninfected controls [104].

#### Risk factors for long COVID

Several risk factors associated with the occurrence of post-infectious sequelae have been identified. As is evident from several studies, long COVID has been seen more frequently in females than in males [105–116]. An important predictor of the development of long COVID in survivors is the older age of the patient [105, 108, 111, 116]. However, there are contradictory reports of the rate of long COVID being slightly higher in young adults [110, 115]. Several pre-existing conditions that are associated with long COVID have been identified, such as obesity, chronic pulmonary disease, alcohol or tobacco consumption, constitutional neuropsychiatric symptoms, and others, such as hypertension, diabetes, asthma or chronic obstructive pulmonary disease (COPD), immunological disorders, hematological disorders, and malignancies [105, 106, 108, 109, 112, 114–116].

The course of acute COVID-19 infection also affects the probability of developing long COVID. In particular, a severe acute illness, longer hospitalization, and ICU stay are important predictors of the occurrence of long COVID [105, 107, 109–111, 113, 114, 116, 117]. The occurrence of neurological complications, such as myalgia, tachycardia, dyspnoea, chest pain, congestion, and depression during acute infection is a predictor of long COVID [105, 107, 112, 117]. Administration of corticosteroids, antibiotics, or intravenous immunoglobulins has also been found to be associated with increased incidence of long COVID [105, 112, 116, 117].

Which variant of SARS-CoV-2 caused the initial acute infection does not appear to affect the development of long COVID, as demonstrated in a study involving 57,727 SARS-CoV-2-positive individuals in which the incidence of long COVID was compared between patients infected with the Delta and Omicron variants [118].

#### Neurological manifestations of long COVID

Generalized fatigue, weakness, or malaise have consistently been found to be the most common sequelae of SARS-CoV-2 infection [71, 94, 119]. Additionally, a wide array of chronic neurological complications have been reported, involving the central nervous system (for example, headache, fatigue, confusion/‘brain fog’, insomnia, or cognitive impairment, and neuropsychiatric manifestations such as depression and anxiety, dizziness, or dysautonomia) as well as the peripheral nervous system (for example, sensorimotor deficits, myopathies, muscle weakness, myalgias, disturbances in taste and/or smell, or sensorineural hearing loss/tinnitus) [12]. The wide spectrum of reported neurological and psychiatric symptoms and their variable prevalence rates seen in PACS are shown in Online Resource 1.

The progression of post-COVID sequelae over time was described in a meta-analysis of 63 studies, which reported that, from 3 to 6 months postinfection, fatigue (32%), dyspnoea (25%), sleep disorder (24%), and difficulty in concentrating (22%) were the most prevalent symptoms, while effort intolerance (45%), fatigue (36%), sleep disorder (29%), and dyspnoea (25%) were the predominant symptoms observed between 6 and 9 months postinfection. Similarly, between 9 and 12 months postinfection, fatigue (37%) and dyspnoea (21%) were the predominant symptoms. Fatigue continued to persist in 41% of the patients studied beyond 12 months [120]. Consistent with these findings, a cohort study of 121 hospitalized COVID-19 patients showed that fatigue was the most commonly reported symptom (reported by 50% of the patients), followed by dyspnoea (42%) and memory dysfunction (34%). Several other symptoms were also observed, including confusion, paresthesia, anxiety, depression, disturbed sleep, and muscle pain [100].

The high prevalence rates of neurological sequelae of COVID-19 present a major public health challenge. There is a pressing need to standardize the definition and diagnostic criteria for PACS and conduct large-scale prospective studies in order to better understand these symptoms and their risk factors. This will also help physicians to better care for these patients and possibly prevent the occurrence of further complications [71].

## Conclusions

This review highlights neurological involvement in SARS-CoV-2 infection and the many neurological presentations seen during and after the course of the disease. A wide spectrum of neurological syndromes has been reported in COVID-19 patients. The probable pathogenesis of the COVID-19-associated neurological disorders is multifaceted. Across the wide array of these neurological sequelae, there lies a common element of neuroinvasion and systemic inflammation. A few of the well-studied pathogenetic mechanisms include hypoxia, overproduction of cytokines, microvascular pathology, and glial cell dysfunction. Acute neurological manifestations are mostly nonspecific. Post-acute neurological manifestations are diverse, not well defined, and may occur individually or together with other multiorgan dysfunctions. The data pertaining to long COVID are constantly evolving and need to be screened more thoroughly for a better understanding of the disease process and outcome.

It is expected that this review will help healthcare professionals to be aware of the wide constellation of neurological manifestations following SARS-CoV-2 infection for appropriate diagnosis and management and that this will reduce the morbidity in this cohort of patients. Further studies are needed for establishing a definitive association of such symptoms with COVID-19 and also for a better comprehension of the underlying pathophysiological mechanisms.

## Electronic Supplementary Material

Below is the link to the electronic supplementary material

Supplementary Material 1

## Data Availability

The data used for this review are publicly available from the sources described in the methods section.
