# Supplementary material for: SARS-CoV-2 and the nervous system: current perspectives
Source: Arch Virol. 2023 Jun 1;168(6):171. doi: 10.1007/s00705-023-05801-x (PMC10232347; doi:10.1007/s00705-023-05801-x)
Supplement: Supplementary file 1 — Supplementary Material 1 [file 705_2023_5801_MOESM1_ESM.pdf]

SARS-CoV-2 and the Nervous System: Current Perspectives

Archives of Virology, Springer

Amrita Pattanaik [1,*], Sushma Bhandarkar B [1], Lonika Lodha [2], Srilatha Marate [1]

[1] Manipal Institute of Virology, Manipal Academy of Higher Education (MAHE), Manipal, Karnataka, India. PIN-576104

[2] Department of Neurovirology, National Institute of Mental Health and Neurosciences (NIMHANS), Bengaluru, Karnataka, India. PIN-560029

[*]Correspondence: amrita.pattanaik@manipal.edu

Table: The wide spectrum of reported neurological and psychiatric manifestations with the prevalence rates

**Year of publication**	**Location of study**	**Sample Size for followup**	**Point of follow-up**	**Fatigue**	**Confusion or brain fog or mental slowness**	**Stress or anxiety**	**Taste dysfunction**	**Smell dysfunction**	**Headache**	**Insomnia or sleep difficulties**	**Dizziness or light headedness or vertigo**	**Vision changes**	**Tinnitus**	**Attention Disorder /Cognitive/memory/concentration impairment**	**Stroke**	**Incontinence (fecal or urinary)**	**Functional impairment**	**Hearing loss**	**Sensitivity disorder**	**Intracranial hemorrhage**	**GBS**	**Movement disturbances**	**Gait disturbances**	**Postural instability or falls**	**Swallowing difficulty**	**Myalgia**	**Autoimmune encephalitis**	**Parasthesia or numbness or tingling**	**Reference**
2020	Italy	143	mean of 60.3 (SD, 13.6) days post onset	53.1			~10	~15	~8		~5																		[1]
2020	US	274	14-21 days post onset	71	~20		~25	~20	61																				[2]
2020	UK	68 (from wards)	mean 48 days (range 29-71) post discharge	60.3										16.2		Bowel 2.9, Bladder 8.8									5.9				[3]

Year of publication	Location of study	Sample Size for followup	Point of follow-up	Fatigue	Confusion or brain fog or mental slowness	Stress or anxiety	Taste dysfunction	Smell dysfunction	Headache	Insomnia or sleep difficulties	Dizziness or light headedness or vertigo	Vision changes	Tinnitus	Attention Disorder /Cognitive/memory/concentration impairment	Stroke	Incontinence (fecal or urinary)	Functional impairment	Hearing loss	Sensitivity disorder	Intracranial hemorrhage	GBS	Movement disturbances	Gait disturbances	Postural instability or falls	Swallowing difficulty	Myalgia	Autoimmune encephalitis	Parasthesia or numbness or tingling	Reference
2020	UK	32 (from ICU)	mean 48 days (range 29-71) post discharge	72										34.4		Bowel 3.1, bladder 12.5									12.5				[3]
2020	Netherlands and Belgium	2113	mean period of 79±17 days post onset	87			11	13	38		27	12																	[4]
2021	US	303	median 63 (IQR 53, 87.5) days post onset	37.5	30.8	30.8	26.4		20.7	22.1	12.5	6.3	9.1																[5]
2021	US	1733	median 186 (175-199) days post onset	63		23	7	11	2	26	6																		[6]

Year of publication	Location of study	Sample Size for followup	Point of follow-up	Fatigue	Confusion or brain fog or mental slowness	Stress or anxiety	Taste dysfunction	Smell dysfunction	Headache	Insomnia or sleep difficulties	Dizziness or light headedness or vertigo	Vision changes	Tinnitus	Attention Disorder /Cognitive/memory/concentration impairment	Stroke	Incontinence (fecal or urinary)	Functional impairment	Hearing loss	Sensitivity disorder	Intracranial hemorrhage	GBS	Movement disturbances	Gait disturbances	Postural instability or falls	Swallowing difficulty	Myalgia	Autoimmune encephalitis	Parasthesia or numbness or tingling	Reference
2021	China	538	median 97 (range 91 to 166) days post discharge	28.3		6.5				17.7	2.6																		[7]
2021	UK	110	Median 90 days post onset	39				10	~5	24																			[8]
2021	China	1276	median 185 days post onset	52			7	11	2	27	6																		[9]
2021	China	1276	median 349 days post onset	20			3	4	5	17	5																		[9]
2021	US	236379	6 months			23.98				5.4					2.1					0.5	0.08								[10]
2021	China	932	3 months	1.8			0.8	0.5																					[11]
2021	Italy	91	30 days post discharge	52		21	14			31																			[12]

Year of publication	Location of study	Sample Size for followup	Point of follow-up	Fatigue	Confusion or brain fog or mental slowness	Stress or anxiety	Taste dysfunction	Smell dysfunction	Headache	Insomnia or sleep difficulties	Dizziness or light headedness or vertigo	Vision changes	Tinnitus	Attention Disorder /Cognitive/memory/concentration impairment	Stroke	Incontinence (fecal or urinary)	Functional impairment	Hearing loss	Sensitivity disorder	Intracranial hemorrhage	GBS	Movement disturbances	Gait disturbances	Postural instability or falls	Swallowing difficulty	Myalgia	Autoimmune encephalitis	Parasthesia or numbness or tingling	Reference
2021	France	478	median 113 days post discharge	31.1	10.1			6	5.5					17.5														12.1	[13]
2021	Germany	442	median 131 days (IQR 112–149	9.7			11.1	12.4																					[14]
2021	Germany	353	median 207 days (IQR 187–234	14.7			11	14.7																					[14]
2021	Spain	797	6 months post discharge	22.1	2.6	6.8	7.2		5.3	4.9	1.9	4.6										3.4						3.4	[15]
2021	China	796	6 months post discharge	25.3						23.2																			[16]
2021	Italy	165	6 months post discharge	33.9	13.3	26.7	16.4			31.5		19.5		31.5		13.9						10.3	10.9	8.5	6.1			18.8	[17]
2021	Norway	312	6 months post illness	37			25							26															[18]

Year of publication	Location of study	Sample Size for followup	Point of follow-up	Fatigue	Confusion or brain fog or mental slowness	Stress or anxiety	Taste dysfunction	Smell dysfunction	Headache	Insomnia or sleep difficulties	Dizziness or light headedness or vertigo	Vision changes	Tinnitus	Attention Disorder /Cognitive/memory/concentration impairment	Stroke	Incontinence (fecal or urinary)	Functional impairment	Hearing loss	Sensitivity disorder	Intracranial hemorrhage	GBS	Movement disturbances	Gait disturbances	Postural instability or falls	Swallowing difficulty	Myalgia	Autoimmune encephalitis	Parasthesia or numbness or tingling	Reference
2021	China	2433	1 year post illness	27.7		10.4	1.4	1.3	2.3		3.3																		[19]
2021	Spain	277	median 77 days (IQR 72–85) post onset	34.8			21.4		17.8			5.4		15.2															[20]
2021	US	177	median 169 (range 31-300) days post onset	13.6	2.3		13.6																						[21]
2021	Italy	599	median 191 (IQR 172-204) days post onset	13.1			10.4		2.7			0.3																	[22]

Year of publication	Location of study	Sample Size for followup	Point of follow-up	Fatigue	Confusion or brain fog or mental slowness	Stress or anxiety	Taste dysfunction	Smell dysfunction	Headache	Insomnia or sleep difficulties	Dizziness or light headedness or vertigo	Vision changes	Tinnitus	Attention Disorder /Cognitive/memory/concentration impairment	Stroke	Incontinence (fecal or urinary)	Functional impairment	Hearing loss	Sensitivity disorder	Intracranial hemorrhage	GBS	Movement disturbances	Gait disturbances	Postural instability or falls	Swallowing difficulty	Myalgia	Autoimmune encephalitis	Parasthesia or numbness or tingling	Reference
2021	Norway	676	mean 132 days (SD 35 days) after testing	23			14		13																	8			[23]
2021	Colombia	100	median 219 days (IQR: 143–258) post onset	34			15	11	45	1	31	21	23	36												42		38	[24]
2021	Bangladesh	355	Continuous followup for atleast 1 month post recovery	33				2	3.4	3.9	2.3		0.3													0.6			[25]

Year of publication	Location of study	Sample Size for followup	Point of follow-up	Fatigue	Confusion or brain fog or mental slowness	Stress or anxiety	Taste dysfunction	Smell dysfunction	Headache	Insomnia or sleep difficulties	Dizziness or light headedness or vertigo	Vision changes	Tinnitus	Attention Disorder /Cognitive/memory/concentration impairment	Stroke	Incontinence (fecal or urinary)	Functional impairment	Hearing loss	Sensitivity disorder	Intracranial hemorrhage	GBS	Movement disturbances	Gait disturbances	Postural instability or falls	Swallowing difficulty	Myalgia	Autoimmune encephalitis	Parasthesia or numbness or tingling	Reference
2021	US	273,618	6 months post infection	12.82	22.82				8.6					7.8												3.2			[26]
2021	UK	327	Median 222 days post covid	83																									[27]
2022	UK	144	Mean of 143.3 days post onset	53.5			6.3	6.9		14.6				18															[28]

Year of publication	Location of study	Sample Size for followup	Point of follow-up	Fatigue	Confusion or brain fog or mental slowness	Stress or anxiety	Taste dysfunction	Smell dysfunction	Headache	Insomnia or sleep difficulties	Dizziness or light headedness or vertigo	Vision changes	Tinnitus	Attention Disorder /Cognitive/memory/concentration impairment	Stroke	Incontinence (fecal or urinary)	Functional impairment	Hearing loss	Sensitivity disorder	Intracranial hemorrhage	GBS	Movement disturbances	Gait disturbances	Postural instability or falls	Swallowing difficulty	Myalgia	Autoimmune encephalitis	Parasthesia or numbness or tingling	Reference
2022	US	156	median 351 (range 82-457) post onset	82	67				60	59	54	~25	~30	~50															[29]
2022	Meta-analysis	10530		37	32	23	12	10	15	31				28												17			[30]
2022	Poland	2218	3 months post infection	35.6	12.1			4.4	29.8																				[31]
2022	Korea	900	6 months post infection	25.3	12	24.1	6.6	8.7	12.4	21.2	14.9		6.6	25.3				2.5											[32]

Year of publication	Location of study	Sample Size for followup	Point of follow-up	Fatigue	Confusion or brain fog or mental slowness	Stress or anxiety	Taste dysfunction	Smell dysfunction	Headache	Insomnia or sleep difficulties	Dizziness or light headedness or vertigo	Vision changes	Tinnitus	Attention Disorder /Cognitive/memory/concentration impairment	Stroke	Incontinence (fecal or urinary)	Functional impairment	Hearing loss	Sensitivity disorder	Intracranial hemorrhage	GBS	Movement disturbances	Gait disturbances	Postural instability or falls	Swallowing difficulty	Myalgia	Autoimmune encephalitis	Parasthesia or numbness or tingling	Reference
2022	Korea	241	12 months	16.2	7.1	16.2	3.3	6.2	4.6	13.3	10.4		5.4	22.4				2.1											[32]
2022	Switzerland	1807 Covid-pos, 882 Covid-neg	Mean 83 days post positive test (mean 84 post negative test)				2.6	3.5		2.4																			[33]
2022	India	667	6 months	32.2			4.9	5.1	9.9	11.1																			[34]
2022	Bangladesh	659	Unclear	42			29							18															[35]

Year of publication	Location of study	Sample Size for followup	Point of follow-up	Fatigue	Confusion or brain fog or mental slowness	Stress or anxiety	Taste dysfunction	Smell dysfunction	Headache	Insomnia or sleep difficulties	Dizziness or light headedness or vertigo	Vision changes	Tinnitus	Attention Disorder /Cognitive/memory/concentration impairment	Stroke	Incontinence (fecal or urinary)	Functional impairment	Hearing loss	Sensitivity disorder	Intracranial hemorrhage	GBS	Movement disturbances	Gait disturbances	Postural instability or falls	Swallowing difficulty	Myalgia	Autoimmune encephalitis	Parasthesia or numbness or tingling	Reference
2022	Netherlands	246 ICU admitted COVID pt.	1 yr post discharge	56.1		17.9	6.1	6.9	5.3		11.5	4.9		16.2		3.7									1.2	21.3		20.6	[36]
2022	Saudi Arabia	504	>12 weeks post covid	19.6		~4	~8	~14	~8																				[37]
2023	Italy	103	Mean 243 days post infection	56.3			56.3		45.6	29.1	6.8			45															[38]
2023	Ukraine	332	>4 weeks post infection	90	25			70		70																			[39]

Year of publication	Location of study	Sample Size for followup	Point of follow-up	Fatigue	Confusion or brain fog or mental slowness	Stress or anxiety	Taste dysfunction	Smell dysfunction	Headache	Insomnia or sleep difficulties	Dizziness or light headedness or vertigo	Vision changes	Tinnitus	Attention Disorder /Cognitive/memory/concentration impairment	Stroke	Incontinence (fecal or urinary)	Functional impairment	Hearing loss	Sensitivity disorder	Intracranial hemorrhage	GBS	Movement disturbances	Gait disturbances	Postural instability or falls	Swallowing difficulty	Myalgia	Autoimmune encephalitis	Parasthesia or numbness or tingling	Reference
2023	Korea	440	>4 weeks post infection		38.6				31.1		29			23.6															[40]
2023	Sweden	204,805	270 (96% participants) and 360 (59% participants) days post positive test	29 in non-hospitalized; 22 in hospitalized, 29 in ICU																									[41]
2023	Palestine	669	90 days post covid	14.9	2.4		7.3	4.6	9	6.3	1.9																		[42]
2022	Meta-analysis	55	Mean: 33.2 days									12.7			16.4						21.8								[43]

1. Patients showing neurological manifestations have been represented in percentages.
2. Columns of percentages for taste and smell dysfunction have been merged wherever they have been reported as single entity.
3. ~ used for approximate percentages where exact numerical data was not available in the study (have been taken from graphs)

References:

1. Carfì A, Bernabei R, Landi F, for the Gemelli Against COVID-19 Post-Acute Care Study Group (2020) Persistent Symptoms in Patients After Acute COVID-19. JAMA 324:603–605. https://doi.org/10.1001/jama.2020.12603

2. Tenforde MW (2020) Symptom Duration and Risk Factors for Delayed Return to Usual Health Among Outpatients with COVID-19 in a Multistate Health Care Systems Network — United States, March–June 2020. MMWR Morb Mortal Wkly Rep 69:. https://doi.org/10.15585/mmwr.mm6930e1

3. Halpin SJ, McIvor C, Whyatt G, et al (2021) Postdischarge symptoms and rehabilitation needs in survivors of COVID-19 infection: A cross-sectional evaluation. Journal of Medical Virology 93:1013–1022. https://doi.org/10.1002/jmv.26368

4. Goërtz YMJ, Van Herck M, Delbressine JM, et al (2020) Persistent symptoms 3 months after a SARS-CoV-2 infection: the post-COVID-19 syndrome? ERJ Open Res 6:00542–02020. https://doi.org/10.1183/23120541.00542-2020

5. Bell ML, Catalfamo CJ, Farland LV, et al (2021) Post-acute sequelae of COVID-19 in a non-hospitalized cohort: Results from the Arizona CoVHORT. PLoS One 16:e0254347. https://doi.org/10.1371/journal.pone.0254347

6. Chopra V, Flanders SA, O'Malley M, et al (2021) Sixty-Day Outcomes Among Patients Hospitalized With COVID-19. Ann Intern Med 174:576–578. https://doi.org/10.7326/M20-5661

7. Xiong Q, Xu M, Li J, et al (2021) Clinical sequelae of COVID-19 survivors in Wuhan, China: a single-centre longitudinal study. Clinical Microbiology and Infection 27:89–95. https://doi.org/10.1016/j.cmi.2020.09.023

8. Arnold DT, Hamilton FW, Milne A, et al (2021) Patient outcomes after hospitalisation with COVID-19 and implications for follow-up: results from a prospective UK cohort. Thorax 76:399–401. https://doi.org/10.1136/thoraxjnl-2020-216086

9. Huang L, Yao Q, Gu X, et al (2021) 1-year outcomes in hospital survivors with COVID-19: a longitudinal cohort study. The Lancet 398:747–758. https://doi.org/10.1016/S0140-6736(21)01755-4

10. Taquet M, Geddes JR, Husain M, et al (2021) 6-month neurological and psychiatric outcomes in 236 379 survivors of COVID-19: a retrospective cohort study using electronic health records. The Lancet Psychiatry 8:416–427. https://doi.org/10.1016/S2215-0366(21)00084-5

11. Sun LL, Wang J, Wang YS, et al (2021) Symptomatic features and prognosis of 932 hospitalized patients with coronavirus disease 2019 in Wuhan. Journal of Digestive Diseases 22:271–281. https://doi.org/10.1111/1751-2980.12983

12. Boari GEM, Bonetti S, Braglia-Orlandini F, et al (2021) Short-Term Consequences of SARS-CoV-2-Related Pneumonia: A Follow Up Study. High Blood Press Cardiovasc Prev 28:373–381. https://doi.org/10.1007/s40292-021-00454-w

13. Writing Committee for the COMEBAC Study Group, Morin L, Savale L, et al (2021) Four-Month Clinical Status of a Cohort of Patients After Hospitalization for COVID-19. JAMA 325:1525–1534. https://doi.org/10.1001/jama.2021.3331

14. Augustin M, Schommers P, Stecher M, et al (2021) Post-COVID syndrome in non-hospitalised patients with COVID-19: a longitudinal prospective cohort study. The Lancet Regional Health - Europe 6:100122. https://doi.org/10.1016/j.lanepe.2021.100122

15. Romero-Duarte Á, Rivera-Izquierdo M, Guerrero-Fernández de Alba I, et al (2021) Sequelae, persistent symptomatology and outcomes after COVID-19 hospitalization: the ANCOHVID multicentre 6-month follow-up study. BMC Medicine 19:129. https://doi.org/10.1186/s12916-021-02003-7

16. Shang YF, Liu T, Yu JN, et al (2021) Half-year follow-up of patients recovering from severe COVID-19: Analysis of symptoms and their risk factors. Journal of Internal Medicine 290:444–450. https://doi.org/10.1111/joim.13284

17. Pilotto A, Cristillo V, Cotti Piccinelli S, et al (2021) Long-term neurological manifestations of COVID-19: prevalence and predictive factors. Neurol Sci 42:4903–4907. https://doi.org/10.1007/s10072-021-05586-4

18. Blomberg B, Mohn KG-I, Brokstad KA, et al (2021) Long COVID in a prospective cohort of home-isolated patients. Nature Medicine 27:1607–1613. https://doi.org/10.1038/s41591-021-01433-3

19. Zhang X, Wang F, Shen Y, et al (2021) Symptoms and Health Outcomes Among Survivors of COVID-19 Infection 1 Year After Discharge From Hospitals in Wuhan, China. JAMA Network Open 4:e2127403. https://doi.org/10.1001/jamanetworkopen.2021.27403

20. Moreno-Pérez O, Merino E, Leon-Ramirez J-M, et al (2021) Post-acute COVID-19 syndrome. Incidence and risk factors: A Mediterranean cohort study. Journal of Infection 82:378–383. https://doi.org/10.1016/j.jinf.2021.01.004

21. Logue JK, Franko NM, McCulloch DJ, et al (2021) Sequelae in Adults at 6 Months After COVID-19 Infection. JAMA Network Open 4:e210830–e210830. https://doi.org/10.1001/jamanetworkopen.2021.0830

22. Peghin M, Palese A, Venturini M, et al (2021) Post-COVID-19 symptoms 6 months after acute infection among hospitalized and non-hospitalized patients. Clinical Microbiology and Infection 27:1507–1513. https://doi.org/10.1016/j.cmi.2021.05.033

23. Søraas A, Kalleberg KT, Dahl JA, et al (2021) Persisting symptoms three to eight months after non-hospitalized COVID-19, a prospective cohort study. PLOS ONE 16:e0256142. https://doi.org/10.1371/journal.pone.0256142

24. Anaya J-M, Rojas M, Salinas ML, et al (2021) Post-COVID syndrome. A case series and comprehensive review. Autoimmunity Reviews 20:102947. https://doi.org/10.1016/j.autrev.2021.102947

25. Mahmud R, Rahman MM, Rassel MA, et al (2021) Post-COVID-19 syndrome among symptomatic COVID-19 patients: A prospective cohort study in a tertiary care center of Bangladesh. PLoS One 16:e0249644. https://doi.org/10.1371/journal.pone.0249644

26. Taquet M, Dercon Q, Luciano S, et al (2021) Incidence, co-occurrence, and evolution of long-COVID features: A 6-month retrospective cohort study of 273,618 survivors of COVID-19. PLoS Med 18:e1003773. https://doi.org/10.1371/journal.pmed.1003773

27. Sigfrid L, Drake TM, Pauley E, et al (2021) Long Covid in adults discharged from UK hospitals after Covid-19: A prospective, multicentre cohort study using the ISARIC WHO Clinical Characterisation Protocol. The Lancet Regional Health – Europe 8:. https://doi.org/10.1016/j.lanepe.2021.100186

28. Gautam N, Madathil S, Tahani N, et al (2022) Medium-Term Outcomes in Severely to Critically Ill Patients With Severe Acute Respiratory Syndrome Coronavirus 2 Infection. Clinical Infectious Diseases 74:301–308. https://doi.org/10.1093/cid/ciab341

29. Tabacof L, Tosto-Mancuso J, Wood J, et al (2022) Post-acute COVID-19 Syndrome Negatively Impacts Physical Function, Cognitive Function, Health-Related Quality of Life, and Participation. American Journal of Physical Medicine & Rehabilitation 101:48. https://doi.org/10.1097/PHM.0000000000001910

30. Premraj L, Kannapadi NV, Briggs J, et al (2022) Mid and long-term neurological and neuropsychiatric manifestations of post-COVID-19 syndrome: A meta-analysis. J Neurol Sci 434:120162. https://doi.org/10.1016/j.jns.2022.120162

31. Chudzik M, Babicki M, Kapusta J, et al (2022) Long-COVID Clinical Features and Risk Factors: A Retrospective Analysis of Patients from the STOP-COVID Registry of the PoLoCOV Study. Viruses 14:1755. https://doi.org/10.3390/v14081755

32. Kim Y, Bitna-Ha, Kim S-W, et al (2022) Post-acute COVID-19 syndrome in patients after 12 months from COVID-19 infection in Korea. BMC Infectious Diseases 22:93. https://doi.org/10.1186/s12879-022-07062-6

33. Nehme M, Vetter P, Chappuis F, et al (2022) Prevalence of Post-COVID Condition 12 Weeks After Omicron Infection Compared With Negative Controls and Association With Vaccination Status. Clinical Infectious Diseases. https://doi.org/10.1093/cid/ciac947

34. Raj SVA, Jacob A, Ambu V, et al (2022) Post COVID-19 clinical manifestations and its risk factors among patients in a Northern District in Kerala, India. J Family Med Prim Care 11:5312–5319. https://doi.org/10.4103/jfmpc.jfmpc_131_22

35. Khandker S, Akther A, Syed BH, et al (2022) Post-COVID-19 complications in home and hospital-based care: A study from Dhaka city, Bangladesh. Frontiers in Rehabilitation Sciences 3:

36. Heesakkers H, van der Hoeven JG, Corsten S, et al (2022) Clinical Outcomes Among Patients With 1-Year Survival Following Intensive Care Unit Treatment for COVID-19. JAMA 327:559–565. https://doi.org/10.1001/jama.2022.0040

37. Alghamdi SA, Alfares MA, Alsulami RA, et al (2022) Post-COVID-19 Syndrome: Incidence, Risk Factor, and the Most Common Persisting Symptoms. Cureus 14:e32058. https://doi.org/10.7759/cureus.32058

38. Taruffi L, Muccioli L, Mitolo M, et al (2023) Neurological Manifestations of Long COVID: A Single-Center One-Year Experience. Neuropsychiatr Dis Treat 19:311–319. https://doi.org/10.2147/NDT.S387501

39. Muzyka I, Yakhnytska M, Savytska M, Zayachkivska O (2023) Long COVID prevalence and physiology-centered risks: population-based study in Ukraine. Inflammopharmacology. https://doi.org/10.1007/s10787-023-01177-1

40. Jung YH, Ha E-H, Park J, et al (2023) Neurological and Psychiatric Manifestations of Post-COVID-19 Conditions. J Korean Med Sci 38:e83. https://doi.org/10.3346/jkms.2023.38.e83

41. Hedberg P, Granath F, Bruchfeld J, et al (2023) Post COVID-19 condition diagnosis: A population-based cohort study of occurrence, associated factors, and healthcare use by severity of acute infection. J Intern Med 293:246–258. https://doi.org/10.1111/joim.13584

42. Abu Hamdh B, Nazzal Z (2023) A prospective cohort study assessing the relationship between long-COVID symptom incidence in COVID-19 patients and COVID-19 vaccination. Scientific Reports 13:4896. https://doi.org/10.1038/s41598-023-30583-2

43. Ahmed JO, Ahmad SA, Hassan MN, et al (2022) Post COVID-19 neurological complications; a meta-analysis. Ann Med Surg (Lond) 76:103440. https://doi.org/10.1016/j.amsu.2022.103440
